# Redefining Unilateral Pulmonary Edema after Mitral Valve Surgery on Chest X-ray Imaging Using the RALE Scoring System

**DOI:** 10.3390/jcm12186043

**Published:** 2023-09-19

**Authors:** Karim Mostafa, Carmen Wolf, Svea Seehafer, Agreen Horr, Nina Pommert, Assad Haneya, Georg Lutter, Thomas Pühler, Marcus Both, Olav Jansen, Patrick Langguth

**Affiliations:** 1 Department of Radiology and Neuroradiology, University Hospital Schleswig-Holstein, Campus Kiel, 24105 Kiel, Germany; carmen.wolf02@uksh.de (C.W.); svea.seehafer@uksh.de (S.S.); agreen.horr@uksh.de (A.H.); marcus.both@uksh.de (M.B.); olav.jansen@uksh.de (O.J.); patrick.langguth@uksh.de (P.L.); 2 Department of Cardiovascular Surgery, University Hospital Schleswig-Holstein, Campus Kiel, 24105 Kiel, Germany; nina.pommert@uksh.de (N.P.); assad.haneya@uksh.de (A.H.); georg.lutter@uksh.de (G.L.); 3 Department of Cardiovascular Surgery, University Hospital Schleswig-Holstein, Campus Luebeck, 23562 Lübeck, Germany; thomas.puehler@uksh.de

**Keywords:** unilateral pulmonary edema (UPE), Radiographic Assessment of Lung Edema (RALE), mitral valve surgery (MVS), mitral valve surgery complications

## Abstract

**Introduction**: Unilateral pulmonary edema (UPE) is a potential complication after mitral valve surgery (MVS), and its cause is not yet fully understood. Definitions are inconsistent, and previous studies have reported wide variance in the incidence of UPE. This research aims at the evaluation of the Radiographic Assessment of Lung Edema (RALE) score concerning assessment of UPE after MVS in order to provide an accurate and consistent definition of this pathology. **Methods and Results:** Postoperative chest X-ray images of 676 patients after MVS (minimally invasive MVS, *n* = 434; conventional MVS, *n* = 242) were retrospectively analyzed concerning presence of UPE. UPE was diagnosed only after exclusion of other pathologies up until the eighth postoperative day. RALE values were calculated for each patient. ROC analysis was performed to assess diagnostic performance. UPE was diagnosed in 18 patients (2.8%). UPE occurred significantly more often in the MI-MVS group (*p* = 0.045; MI-MVS *n* = 15; C-MVS *n* = 3). Postoperative RALE values for the right hemithorax (Q1 + Q2) > 12 and the right-to-left RALE difference ((Q1 + Q2) − (Q3 + Q4)) > 13 provide a sensitivity of up to 100% and 94.4% and a specificity of up to 88.4% and 94.2% for UPE detection. **Conclusion**: The RALE score is a practical tool for assessment of chest X-ray images after MVS with regard to UPE and provides a clear definition of UPE. In addition, it enables objective comparability when assessing of the postoperative course. The given score thresholds provide a sensitivity and specificity of up to 94%. Further, UPE after MVS seems to be a rather rare pathology with an incidence of 2.6%.

## 1. Introduction

Unilateral pulmonary edema (UPE) is a potentially life-threatening complication observed in the postoperative period following mitral valve surgery (MVS). MVS includes mitral valve reconstruction or replacement as established therapies for mitral regurgitation.

MVS includes two main surgical techniques that differ in their approach to the mitral valve, but both require intraoperative cardiopulmonary bypass. The conventional median sternotomy approach (C-MVS) is associated with a high risk of intra- and postoperative complications, including bleeding in need of transfusion [1]. Alternatively, MVS can be performed minimally invasively (MI-MVS) via a mini-thoracotomy at the right lateral hemithorax to reduce surgical trauma. Previous studies have shown that MI-MVS has a comparatively low mortality rate, and similar perioperative outcomes have been reported [2,3]. In addition, MI-MVS has been documented to have a shorter hospital length of stay, less postoperative pain and better cosmetic results [2,3]. However, a known complication after MI-MVS is the development of UPE, most commonly affecting the right lung. Clinical signs of this complication can be evident in varying degree, manifesting themselves as respiratory or hemodynamic deterioration. In severe cases, oxygenation impairment and cardiac failure can result in requirement of extracorporeal life support, which is associated with an increased mortality rate [4,5]. However, the pathogenesis of UPE after MVS is not well understood. Several studies have discussed different pathogenetic pathways, with pulmonary re-expansion edema being considered the most likely reason; however, inflammatory processes, pulmonary microvasculature abnormalities, and an association with prolonged cardiopulmonary bypass (CPB) have been mentioned [6,7]. If CPB time were to have an influence on the development of UPE, it would seem reasonable to assume that it would also occur after C-MVS. However, to our knowledge, the occurrence of UPE after C-MVS has not yet been mentioned in the literature. Finally, the exact causes and pathogenesis of UPE remain unclear. The reported incidence of UPE after MI-MVS varies from 1.6% up to 28% [4,5,8] (Table S1). This may be due to differences in the patient population, surgical expertise and procedures, or postoperative management. However, the main reason for this discrepancy may be the lack of precise diagnostic criteria for UPE. UPE is primarily diagnosed upon chest X-ray imaging, and previous studies have reported several diagnostic features, including unilateral opacities > 20% in the first 24 h after surgery, as highly suggestive of UPE. However, in the immediate postoperative setting after MI-MVS, various conditions associated with unilateral opacities may mimic UPE and be erroneously defined as such. This may lead to a significant diagnostic bias and distort the incidence of UPE [9,10]. In this study, we investigated the incidence of UPE after MI-MVS and C-MVS on chest X-ray, considering all available imaging studies completed within the first eight postoperative days, and used the Radiographic Assessment of Lung Edema (RALE) score for quantitative assessment. The RALE score was originally validated to assess the severity of lung edema in acute respiratory distress syndrome (ARDS) [11].

## 2. Material and Methods

This study was conducted according to the declaration of Helsinki and was approved by our local institutional ethics board (No. AZ-D-559/18). All patients gave written informed consent before enrolment.

### 2.1. Study Population

All patients who underwent elective MVS at our center between 2008 and 2022 were included in this study. Patients were divided into two groups according to the surgical technique of MVS: conventional approach via a median sternotomy (C-MVS) or minimally invasive approach via a mini-thoracotomy (MI-MVS). Demographic and periprocedural data were collected for each group. All patients in both groups were screened for UPE.

### 2.2. Chest X-ray Analysis and Diagnosis of UPE

**Figure 1:**
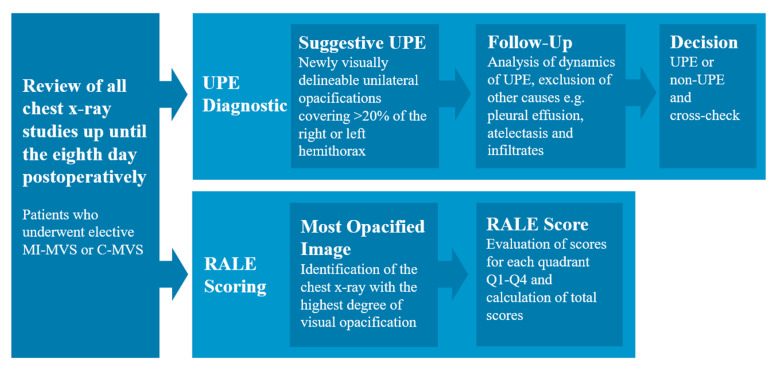
Flowchart for diagnosis of UPE and RALE scoring.

**Figure 2:**
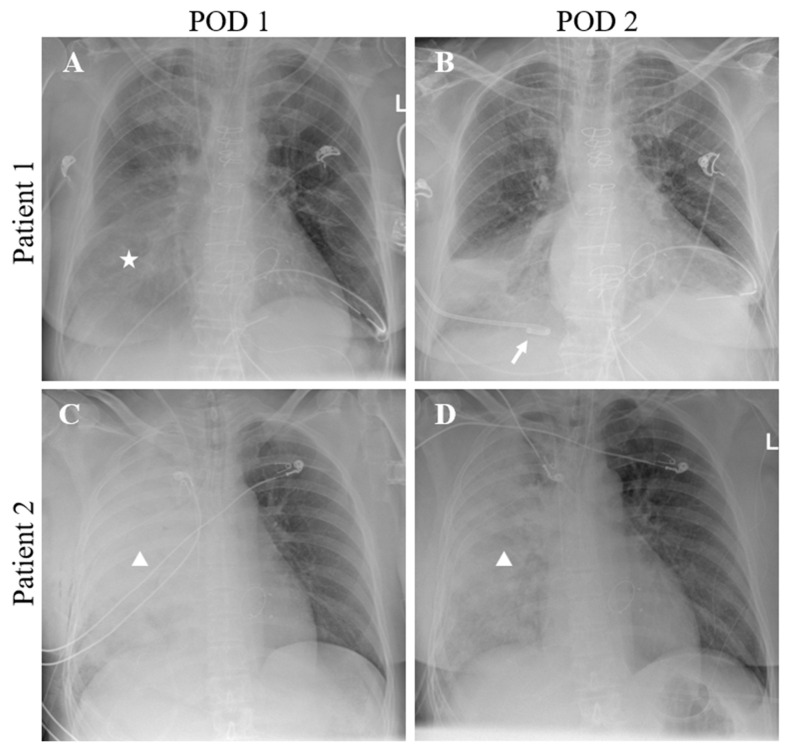
Two different clinical situations after MVS with unilateral opacities on chest X-ray. The first postoperative chest X-ray (**A**) of a patient after MVS shows unilateral opacities of the right hemithorax (star), which resolves after insertion of a pleural drain (Arrow, (**B**)). Therefore, the cause of the opacities can be attributed to pleural effusion with atelectasis rather than UPE. In another patient, chest X-ray on the first (**C**) and second day (**D**) after surgery showed a persisting consolidated unilateral opacity of the right hemithorax (triangles), suggesting UPE.

The chest X-ray images were assessed by two radiologists (Karim Mostafa, Svea Seehafer). According to the clinical standard, all patients received a chest X-ray immediately after surgery and at least once a day during the further postoperative course. The chest X-rays were evaluated until the eighth postoperative day. The diagnosis of UPE was made as follows: All available X-ray studies and thoracic CT imaging up until the eighth postoperative day were reviewed. Preoperative imaging studies were used as a baseline for comparison. Any newly visually delineable unilateral opacifications covering >20% of the right or left hemithorax within eight postoperative days were considered as potential evidence of UPE. Next, all available chest X-ray and thoracic CT imaging studies up until the eighth postoperative day were specifically assessed in order to identify other causes such as pleural effusion, atelectasis or pneumonia, and other causes of unilateral opacities, and to assess the dynamics of these conditions. Finally, only cases where the mentioned conditions were excluded and UPE remained as the only possible explanation for the findings were considered as UPE (Figure 1 and Figure 2). Finally, all cases of UPE were cross-checked between the readers to further ensure the accuracy of the diagnosis and to limit false-positive findings.

The diagnostic assessment of UPE was performed by reviewing all available imaging studies up to the eighth POD to best ensure that other causes of unilateral opacities on chest X-ray were excluded. The RALE score was then calculated for the most opacified chest X-ray within these 8 PODs.

### 2.3. Rale Score Evaluation for Diagnosis of UPE

The RALE score divides the chest X-ray into four quadrants using the vertebral column and the first branch of the left main bronchus as a crosshair boundary [11]. The resulting quadrants are described as Q1–Q4, whereby Q1 represents the upper right quadrant, Q2 represents the lower right quadrant, Q3 represents the upper left quadrant, and Q4 represents the lower left quadrant. Scores are calculated for each quadrant by multiplying the degree of consolidation (0–4) by the density of opacities (1–3) and summing the values for each quadrant (Supplementary Figure S1). The maximum possible score is 12 for each quadrant, 24 for each hemithorax, and 48 for the whole thorax. Score values for the whole thorax, each hemithorax, and each quadrant, and score differences between the right and left hemithorax were noted.

In each patient, the RALE score was calculated for the chest X-ray with the highest degree of visual opacities available within the first eight PODs. Receiver operating characteristic (ROC) analysis was then performed to assess the diagnostic performance of the RALE scoring system in patients with known UPE as defined above. We report cut-off values, sensitivity, specificity, and AUC values for the right-sided RALE score and the difference in score values between the right and left hemithorax ((Q1 + Q2) − (Q3 + Q4)). The cut-off values with the highest possible sensitivity and specificity were determined by Youden’s index.

### 2.4. Statistical Analysis

All statistical analyses were performed using The Jamovi project, version 2.5.5, for Windows (Sydney, Australia, 2021) [12]. Ordinal and non-normally distributed data are presented as medians with interquartiles and were evaluated using the Mann–Whitney U-test as indicated. ROC analysis was performed as described. Statistical significance was set at alpha < 0.05 for all tests.

## 3. Results

### 3.1. Study Population

A total of 676 patients were included in this analysis, of whom 434 underwent MI-MVS and 242 underwent C-MVS. Baseline demographic characteristics are summarized in Table 1. We found significant differences between the two groups in the median age of the patients and the duration of the surgical procedures.

### 3.2. Chest X-ray Analysis and Diagnosis of UPE

In total, approximately 7000 chest X-ray images were evaluated. Overall, we diagnosed UPE in 18/676 (2.6%) cases using the RALE score. In the MI-MVS group, we found 15/434 (3.5%) cases of UPE, compared to 3/242 (1.2%) in the C-MVS group. Thus, UPE was significantly more common in the MI-MVS group than in the C-MVS group (*p* = 0.043, Table 2). The three UPE patients in the C-MVS group were treated for moderate and severe mitral valve insufficiency. In one patient, closure of a patent foramen ovale was performed in the same setting. However, beyond arterial hypertension, coronary and peripheral arterial atherosclerosis, and hypercholesterinemia, no common pathology was present that could be suspected as cause for UPE.

### 3.3. RALE Scoring of Chest X-ray and ROC Analysis Results

In 96% of all patients (*n* = 676), the most opacified chest X-ray was found within the first 72 h postoperatively. The median RALE values for all four quadrants, both hemithoraces, and the RALE values of the whole chest X-ray for the UPE and non-UPE groups are shown in Table 3. In the UPE group, we found significantly higher values in Q1 and Q2, and a greater difference between the right (Q1 + Q2) and left hemithorax (Q3 + Q4) with a *p*-value of <0.001.

### 3.4. ROC Analysis

**Figure 3:**
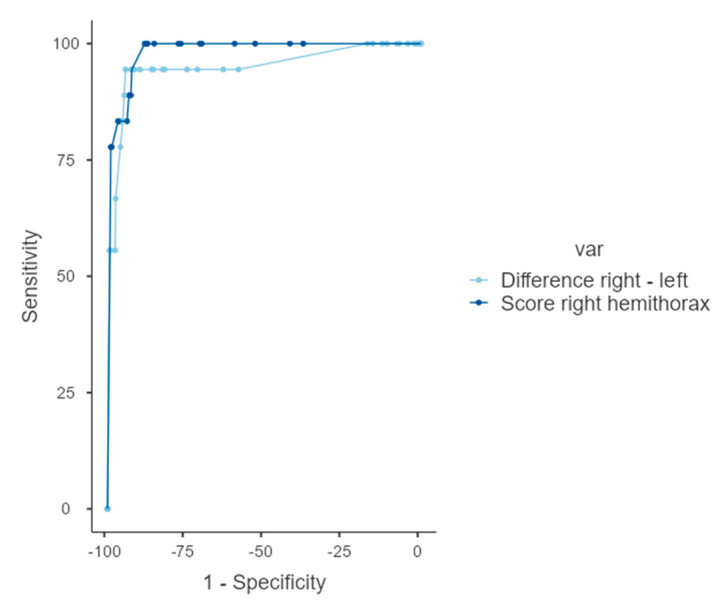
**ROC curves for RALE score analysis**. AUC curves of the difference between the RALE values of both hemithoraces (light blue) and the results of the RALE values of the right hemithorax (dark blue).

Applied as a diagnostic test, the calculated RALE value of the right hemithorax providing the highest sensitivity and specificity for diagnosis of UPE is 12. Here, we report a sensitivity of 100% and specificity of 88.2%. The threshold value for the difference between both hemithoraces was calculated at 13, providing a sensitivity and specificity of 94% (Figure 3, Table 4).

## 4. Discussion

The aim of this retrospective study was to evaluate the RALE score for the targeted diagnostic assessment of UPE on postoperative chest X-ray imaging in patients undergoing MVS. In addition, we investigated the incidence of UPE in patients after MI-MVS and C-MVS.

The main results and conclusions of this study are as follows: (1) The incidence of UPE after MVS is 2.6%. (2) UPE was diagnosed significantly more often in the MI-MVS group than in the C-MVS group. (3) With regard to imaging, UPE cannot be diagnosed by a single chest X-ray, as this would cause a high number of false-positive results. (4) The RALE score is a feasible tool for the objective quantification of opacities on chest X-ray images and is therefore suitable for the diagnostic assessment and follow-up of UPE in patients after MVS. (5) In patients with UPE, sensitivity and specificity of up to 94% can be achieved with the presented RALE score cut-off values.

### 4.1. Diagnosis and Incidence of UPE

In the past decade, studies have reported the incidence of UPE in patients after MVS to range from 1.4% to 25% [5,6,7,13,14,15,16]. As mentioned by Kesävuori et al., the reason for this high range is probably the lack of a standardized objective radiological and clinical definition of this condition [5]. The recent studies by Pühler et al. and Kesävuori et al. defined UPE as unilateral opacities > 20% occurring within the first 24 h after surgery with signs of interstitial thickening, and they also attempted to differentiate between lower and higher grades of UPE by the presence of additional consolidations [5,16]. Both studies reported an incidence of UPE of around 18%. However, as shown by the interobserver agreement of 0.78 reported by Kesävuori et al., these results ultimately depend on the quality of the X-ray image itself and the person reporting the findings. Furthermore, reviewing chest X-ray images only within the first 24 h after MVS may lead to a number of false-positive results, as other pathologies with similar appearance upon imaging cannot be excluded in this short time window. This may artificially increase the incidence of UPE.

In our study, we evaluated all patients’ chest X-rays and other available imaging modalities (e.g., computed tomography) up to the eighth POD to best ensure that pleural effusion, atelectasis, pulmonary hemorrhage, chest wall hematoma, and pneumonia were excluded, as these conditions mimic the imaging findings of UPE upon chest X-ray imaging. This approach enabled us to identify and exclude cases in our study that would otherwise be confused with UPE, and, hence, resulted in an incidence for UPE of 2.6% overall. Moss et al. and Irisawa et al. also reported low incidence of UPE, of 1.4% and 2.5%, respectively, but neither applied a systematic approach to the evaluation of chest X-ray, focusing instead on clinically and radiographically evident cases [13,14]. Thus, Irisawa et al. stated that uneventful cases with ambiguous imaging findings were not included. Our study focused on patients with UPE, which became increasingly likely by excluding other causes of unilateral opacities, by reviewing chest imaging in the 8-day postoperative period in patients after MVS.

The definitive diagnosis of UPE remains the domain of chest imaging, but the currently accepted definition of UPE as postoperative unilateral opacities > 20% is rather vague [5,16]. Although chest X-ray is readily available and provides an overview of the cardiopulmonary status after MVS, there are still limitations in the accurate identification of imaging findings and underlying pathology, particularly due to anterior–posterior image acquisition and the intensive care unit (ICU) setting. In our experience, radiographic imaging acquisition in the ICU setting is often associated with some loss of image quality due to difficult and variable patient positioning and mechanical ventilation with different breathing positions during imaging. This may limit the optimal interpretation of chest X-ray, making it difficult to assess low-grade forms of UPE that are likely to have little or no clinical impact.

### 4.2. RALE Scoring for UPE Assessment

**Figure 4:**
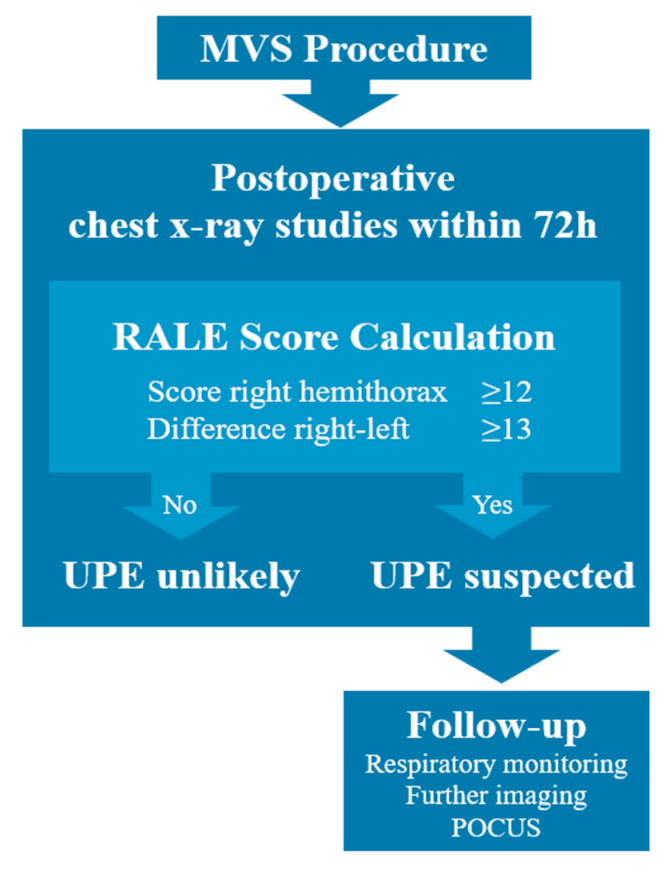
Flowchart for clinical application of the RALE score for assessment of UPE after MVS. (POCUS—point-of-care ultrasound examination).

In our study, we investigated, for the first time, the applicability of the RALE score for the diagnostic assessment of UPE after MVS. The RALE score has been well studied for the diagnosis of ARDS, and studies have suggested RALE threshold values of 9 and 10 for the diagnosis of ARDS, which are consistent with the Horowitz index [11,17,18]. These threshold values cannot be applied to UPE because ARDS is a different disease entity. However, RALE soring allows objective quantification of the extent of opacities and lateral differences in opacities on chest X-ray images, both of which may indicate UPE. Our results show that in patients after MVS, RALE values of ≥12 points for the right hemithorax and/or ≥13 points difference between right and left hemithorax indicate UPE. In our study, we were able to confirm the early occurrence of UPE after MVS, and we therefore recommend that RALE scoring be performed especially within the first 72 h after surgery, as almost all images in our study showed the highest degree of opacifications within this period. Another advantage of the RALE score is that it can be used to assess UPE dynamics over time, even with different reviewers. If RALE values are suspicious for UPE, further diagnostic investigations should be performed. In this regard, it is important to consider additional follow-up imaging studies with different modalities and point-of-care ultrasound examinations to further improve diagnostic quality (Figure 4).

### 4.3. Study Limitations

Although our study provides valuable insights into the diagnostic assessment of UPE and the applicability of the RALE score to this condition, the most important limitation is undoubtedly that our main focus was on the examination of chest X-ray images, whereas clinical data were not assessed. This may limit the generalizability of our findings. In future research, identification of patients according to RALE scoring will provide a reliable selection criterion, and, afterwards, clinical and perioperative parameters can be assessed in an attempt to identify the underlying causes of this pathology and to generate a more comprehensive understanding of UPE. Such a study may provide a clinical definition of UPE, while our work focused on providing a chest X-ray imaging definition. 

## 5. Conclusions

The RALE score is a practicable tool to objectively assess chest X-ray images after MVS for UPE. It allows a differentiated assessment of opacities in both hemithoraces, which is crucial for the diagnosis of UPE. The proposed thresholds of the RALE score for the right hemithorax and the difference between the right and left hemithorax provide a sensitivity and specificity for UPE of up to 94%. In addition, our study suggests that UPE is much less common (2.6%) than previously reported, and more common in MI-MVS than in C-MVS.

## Figures and Tables

**Table 1:** **Characteristics of the study population** with subgroup comparisons. *p*-values were calculated using the Mann–Whitney U-test.

	Overall MVS (*n* = 676)	MI-MVS (*n* = 434)	C-MVS (*n* = 242)	*p*-Value
Demographic data				
Female gender, *n* (%)	275 (40.7)	175 (40.3)	100 (41.3)	0.800
Age (years)	65 [56, 74]	64 [54, 73]	68.5 [59, 75]	<0.001
Weight (kg)	77 [67, 89.3]	75.5 [66, 89]	78 [67, 89.8]	0.308
Height (cm)	174 [167, 181]	174 [167, 182]	172 [166, 180]	0.328
Duration of procedure (minutes)	280 [243, 320]	287 [257, 322]	261 [214, 313]	<0.001

**Table 2:** **Occurrence of UPE** in MI-MVS, C-MVS, and in total. *p*-value was calculated by X^2^-test.

	Overall MVS (*n* = 676)	MI-MVS (*n* = 434)	C-MVS (*n* = 242)	*p*-Value
UPE, *n* (%)	18 (2.6)	15 (3.5)	3 (1.2)	0.043

**Table 3:** **RALE scoring of chest X-ray images**. All calculated scores are presented as median with interquartile range for UPE and non-UPE groups. The *p*-value was calculated using the Mann–Whitney U-test.

	Non-UPE (*n* = 658)	UPE (*n* = 18)	*p*-Value
Scores			
Q1	0 [0, 0]	12 [12, 12]	<0.001
Q2	2 [0, 4]	12 [12, 12]	<0.001
Q3	0 [0, 0]	0 [0, 0]	0.543
Q4	0 [0, 3]	0 [0, 3.5]	0.937
Right hemithorax (Q1 + Q2)	2 [0, 6]	24 [24, 24]	0.001
Left hemithorax (Q3 + Q4)	0 [0, 4]	0 [0, 5]	0.952
RALE score total	4 [0, 10]	24 [24, 24]	<0.001
Difference (Q1 + Q2) to (Q3 + Q4)	0 [0, 4]	24 [16, 24]	<0.001

**Table 4:** **ROC analysis** to determine RALE score criteria for diagnosing UPE.

	Threshold Value	Sensitivity (%)	Specificity (%)	Youden’s Index	AUC
Scores					
Right hemithorax (Q1 + Q2)	12	100	88.2	0.88	0.980
Difference (Q1 + Q2) to (Q3 + Q4)	13	94.44	94.22	0.887	0.949

## Data Availability

The datasets used and/or analyzed during the current study are available from the corresponding author upon reasonable request.

## Supplementary Materials

The following supporting information can be downloaded at: https://www.mdpi.com/article/10.3390/jcm12186043/s1, Figure S1. RALE score calculation example. This patient was diagnosed with UPE in the immediate postoperative period. An exemplary calculation of the RALE score is provided, showing diagnostic features suggestive of UPE (right-sided score > 13 and score difference > 12). Table S1. Previous studies investigating incidence of UPE after MVS.

## Abbreviations

ARDS	Acute respiratory distress syndrome
AUC	Area under the curve
BMI	Body mass index
C-MVS	Conventional mitral valve surgery (median sternotomy)
ICU	Intensive care unit
MIS	Minimally invasive surgery
MVS	Mitral valve surgery
MI-MVS	Minimally invasive mitral valve surgery
POCUS	Point-of-care ultrasound
POD	Postoperative day
RALE	Radiographic assessment of lung edema
ROC	Receiver operating characteristic
UPE	Unilateral pulmonary edema
