# Supplementary material for: Redefining Unilateral Pulmonary Edema after Mitral Valve Surgery on Chest X-ray Imaging Using the RALE Scoring System
Source: J Clin Med. 2023 Sep 19;12(18):6043. doi: 10.3390/jcm12186043 (PMC10532294; doi:10.3390/jcm12186043)
Supplement: Supplementary file 1 [file jcm-12-06043-s001.zip › jcm-2603043-supplementary.pdf]

Redefining Unilateral Pulmonary Edema after Mitral Valve Surgery on Chest X-ray Imaging Using the RALE Scoring System

Supplementary Image

Supplementary Figure:

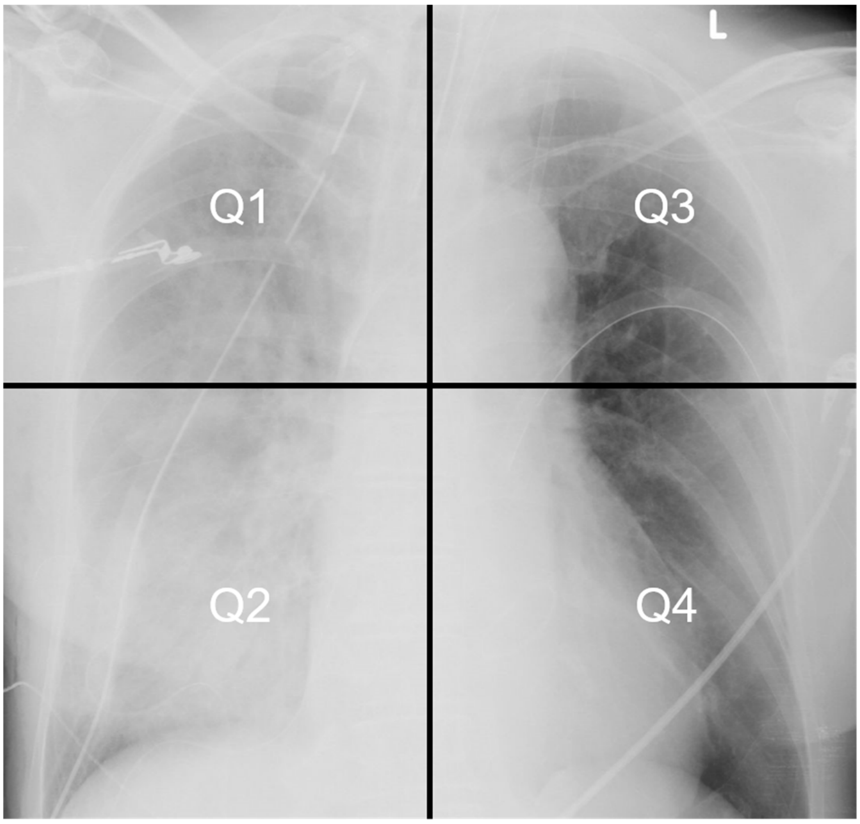

	Q1	Q2	Q3	Q4
Consolidation	4	4	1	2
Opacities	3	3	1	4
Quadrant score	12	12	1	8
Lateral score	24		9	
Overall score	33			
Score difference	15			

Supplementary Figure S1 – RALE score calculation example

This patient was diagnosed with UPE in the immediate postoperative period. An exemplary calculation of the RALE score is provided, showing diagnostic features suggestive of UPE (right-sided score >13 and score difference >12).

Table S1. **Previous studies** investigating incidence of UPE after MVS.

Study	Incidence of UPE	N	Diagnostic criteria for UPE	period
Khalil et al. (2020)	1.6% (UPE)<br>18.8% (UPC)	382 (elective MIV cardiac surgery)	UPC: blurring of central vessel contours, cuffing, Kerley lines<br>UPE: Severe UPC, completely shaded lung parenchyma	2013-2014
Kesävuori et al. (2020)	18.6%	231 (elective MI-MVS)	Opacification of the right lung ≥25%, interstitial thickening, consolidation with air bronchograms	2009-2017
Pühler et al. (2021)	18.3%	629 (MI-MVS)	>20% opacification of the right hemithorax, no direct or indirect signs for atelectasis or other infiltrates occurred within the first 24h postoperatively	2009-2019
López et al. (2022)	28%	93 (MI-MVS)		2015-2017
Tutschka et al. (2015)	25%	277 (cardiac surgery with right Mini thoracotomy + CPB)	Relatively increased opacification of the right versus left hemithorax involving at least 20% of the hemithorax, no atelectasis	2005-2012
Renner et al. (2017)	19.9%	256 (MI-MVS)	>20% opacification of the right hemithorax, no direct or indirect signs for atelectasis or other infiltrates occurred within the first 24h postoperatively	2008-2015
Irisawa et al. (2014)	2.1% (reexpansion pulmonary edema)	381 (minimally invasive cardiac surgery with singles-lung ventilation)	RPO was diagnosed by expert radiologists as a new and apparent UPE on a chest radiography and computed tomography after operation, exclusion of atelectasis	2005-2013
Keyl et al. (2015)	7.9%	484 (minimally invasive cardiac surgery with unilateral lung collapse)	Newly developed UPE which was radiographically evident within 24h after surgery	2008-2013
Moss et al. (2017)	1.2%	1257 (robotic MVS)	Radiologic evidence: multilobed right lung pulmonary infiltration developing within 4h of CPB, with profuse if straw-coloured fluid from the right main stem bronchus, additionally clinical signs were considered	2006-2012
